# Shared and distinct functional connectivity of hippocampal subregions in schizophrenia, bipolar disorder, and major depressive disorder

**DOI:** 10.3389/fpsyt.2022.993356

**Published:** 2022-09-14

**Authors:** Yanzhuo Song, Jingyu Yang, Miao Chang, Yange Wei, Zhiyang Yin, Yue Zhu, Yuning Zhou, Yifang Zhou, Xiaowei Jiang, Feng Wu, Lingtao Kong, Ke Xu, Fei Wang, Yanqing Tang

**Affiliations:** ^1^Department of Psychiatry, First Hospital of China Medical University, Shenyang, China; ^2^Early Intervention Unit, Department of Psychiatry, Affiliated Nanjing Brain Hospital, Nanjing Medical University, Nanjing, China; ^3^Department of Radiology, First Hospital of China Medical University, Shenyang, China

**Keywords:** hippocampal subregion, schizophrenia, bipolar disorder, major depressive disorder, functional connectivity

## Abstract

Schizophrenia (SZ), bipolar disorder (BD), and major depressive disorder (MDD) share etiological and pathophysiological characteristics. Although neuroimaging studies have reported hippocampal alterations in SZ, BD, and MDD, little is known about how different hippocampal subregions are affected in these conditions because such subregions, namely, the cornu ammonis (CA), dentate gyrus (DG), and subiculum (SUB), have different structural foundations and perform different functions. Here, we hypothesize that different hippocampal subregions may reflect some intrinsic features among the major psychiatric disorders, such as SZ, BD, and MDD. By investigating resting functional connectivity (FC) of each hippocampal subregion among 117 SZ, 103 BD, 96 MDD, and 159 healthy controls, we found similarly and distinctly changed FC of hippocampal subregions in the three disorders. The abnormal functions of middle frontal gyrus might be the core feature of the psychopathological mechanisms of SZ, BD, and MDD. Anterior cingulate cortex and inferior orbital frontal gyrus might be the shared abnormalities of SZ and BD, and inferior orbital frontal gyrus is also positively correlated with depression and anxiety symptoms in SZ and BD. Caudate might be the unique feature of SZ and showed a positive correlation with the cognitive function in SZ. Middle temporal gyrus and supplemental motor area are the differentiating features of BD. Our study provides evidence for the different functions of different hippocampal subregions in psychiatric pathology.

## Introduction

Schizophrenia (SZ), bipolar disorder (BD), and major depressive disorder (MDD) are distinct entities according to traditional diagnostic criteria. However, certain issues would appear when applying this set of diagnostic criteria in clinical practice (1, 2). Therefore, there might be no clear distinction among these different diagnoses regarding the involved biological mechanisms. Transdiagnostic studies could provide a deeper understanding of the common and distinct endophenotypes of multiple disorders than traditional single diagnostic studies (3–5). Recently, reports of a high genetic correlation among these three disorders have raised concerns (6, 7) and implied that they may share similar genetic backgrounds. Additionally, molecular and neuroimaging studies have further revealed similar yet still distinct pathophysiological features in SZ, BD, and MDD, suggesting that these three disorders might involve different episodes of a single transdiagnostic continuum of disease (8–14). Thus, exploring the commonalities and distinctions of these three disorders should deepen our understanding of their fundamental underlying mechanisms and might enable the development of precise treatments.

The hippocampus belongs to the limbic system and is involved in memory processing, learning, and emotions. Previous neuroimaging studies described structural and functional abnormalities in the hippocampus in SZ (15–17), BD (18, 19), and MDD (20, 21), suggesting that the hippocampus may be involved in pathophysiological features of these three major psychiatric disorders. However, the hippocampus is a complex region, and can be divided into three distinct subfields according to cytoarchitectonic differences: cornu ammonis (CA), dentate gyrus (DG), and subiculum (SUB) (22). DG receives its input from the entorhinal cortex and connects to CA. SUB is the main target of the outputs of CA (especially CA1) and projects back to the entorhinal cortex (23). The different subregions have different structural foundations and perform different functions in memory and cognition (24). Recent research has revealed that the different hippocampal subregions are differentially affected in SZ, BD, and MDD (25–28). Therefore, we postulate that the hippocampal subregions may reflect some intrinsic features among these three psychiatric disorders.

In this study, we examined the role of hippocampal subregions in SZ, BD, and MDD by exploring the resting functional connectivity (FC) of each subregion with the whole brain in patients and healthy controls. By selecting different hippocampal subregions as core regions, we explored the shared and distinct FC changes among SZ, BD, and MDD and provided candidate endophenotypes and trait measures of the abnormalities of these three disorders.

## Materials and methods

### Participants

This study was conducted at a single site and included a total of 517 individuals aged 13–45 years: 137 with SZ, 109 with BD, 103 with MDD, and 168 matched healthy controls (HC). All participants with SZ, BD, and MDD were recruited from the inpatient and outpatient services at Shenyang Mental Health Center and the Department of Psychiatry, First Hospital of China Medical University, Shenyang, China. HC participants were recruited from the local community *via* advertisements. All participants provided written informed consent after receiving a detailed description of the study (or their parents/guardians did, if they were younger than 18 years old). This study was approved by the Institutional Review Board of China Medical University (approval reference number [2012]25–1) and in accordance with the Declaration of Helsinki.

All SZ, BD, and MDD patients met the diagnostic criteria of the Diagnostic and Statistical Manual of Mental Disorders, Fourth Edition (DSM-IV), and did not meet the criteria for any other Axis I disorder. All patients were evaluated by two trained psychiatrists for the presence or absence of Axis I psychiatric diagnoses, using Structured Clinical Interview for DSM-IV Axis I Disorders (SCID) in patients aged 18 years and older and semi-structured diagnostic interview for the Schedule for Affective Disorders and Schizophrenia for School-Age Children-Present and Lifetime Version (K-SADS-PL) in patients under 18 years of age. Duration of illness was <5 years for the SZ, BD, and MDD groups. HC participants did not have a current or lifetime Axis I disorder or history of psychotic, mood, or other Axis I disorders in first-degree relatives, as determined by a detailed family history.

Participants were excluded if any of the following was present: (1) substance/alcohol abuse or dependence or concomitant major medical disorder, (2) any magnetic resonance imaging (MRI) contraindications, and (3) history of head trauma with loss of consciousness for ≥5 min or any neurological disorder. For all participants, symptom measures were obtained using the 17-item Hamilton Rating Scale for Depression (HAMD-17), Hamilton Rating Scale for Anxiety (HAMA), Young Mania Rating Scale (YMRS), and Brief Psychiatric Rating Scale (BPRS), and cognitive function was evaluated by the Wisconsin Card Sorting Test (WCST), for example, correct responses, categories completed, total errors, perseverative errors, and non-perseverative errors.

### MRI acquisition

MRI data were acquired using a GE Signa HD 3.0-T scanner (General Electric, Milwaukee, WI) with a standard eight-channel head coil at the First Affiliated Hospital of China Medical University, Shenyang, PR China. Functional images were collected using a gradient echo planar imaging (EPI-GRE) sequence. The following parameters were used: TR = 2,000 ms, TE = 30 ms, flip angle = 90°, field of view = 240 × 240 mm^2^, and matrix = 64 × 64. Thirty-five axial slices were collected with a 3 mm thickness, without a gap. The scan lasted 6 min and 40 s, resulting in 200 volumes. Participants were instructed to rest and relax with their eyes closed but to remain awake during scanning.

### Data processing

MRI data were preprocessed using the Statistical Parametric Mapping 8 (SPM8, http://www.fil.ion.ucl.ac.uk/spm) and Data Processing Assistant for R-fMRI (DPARSF, http://www.restfmri.net/forum/DPARSF) toolkits (29). The first 10 time points were discarded due to magnetic saturation effects. The remaining data were slice-time corrected and then realigned to the first volume to correct for head motion. Each participant's head motion was assessed by means of translation/rotation, and exclusion criteria (translation >3 mm, rotation >3° in each direction) were set. A total of 42 subjects were excluded due to head motion, and the remaining sample size was 117 SZ, 103 BD, 96 MDD, and 159 HC (Table 1). Images were normalized to the standard EPI template in Montreal Neurological Institute (MNI) space and resampled to 3 × 3 × 3 mm^3^. Images were spatially smoothed with a 6 mm full width at half maximum (FWHM) Gaussian kernel. The linear regression of nuisance covariates was built based on 24 head motion parameters, cerebro-spinal fluid signal, white matter signal, and linear trend.

**Table 1:** Demographic and clinical characteristics.

	**SZ**	**BD**	**MDD**	**HC**	***t/F/χ2***	***p***
**Demographic characteristics**						
Numbers of subjects	*n* = 117	*n* = 103	*n* = 96	*n* = 159		
Age, years	24.52 (8.948)	25.79 (7.622)	25.33 (9.217)	26.12 (8.048)	0.863^a^	0.46
Years of education	10.63 (2.87)	12.52 (2.94)	11.99 (2.878)	14.42 (3.296)	36.641^a^	<0.001
Gender, Male	52	50	34	74	0.246^a^	0.241
**Clinical characteristics**						
Duration, months	24.21 (38.00)	38.43 (52.04)	18.21 (23.83)	–	6.556^b^	0.003
First episode, yes	78	57	82	–	21.24^b^	<0.001
Medication, yes	74	68	41	–	13.248^b^	0.001
HAMD-17	*n* = 86	*n* = 101	*n* = 94	*n* = 142		
	7.87 (6.957)	11.92 (9.534)	21.6 (9.635)	1.15 (1.66)	57.849^b^	<0.001
HAMA	*n* = 70	*n* = 96	*n* = 81	*n* = 141		
	6.8 (7.814)	8.41 (8.302)	17.11 (9.6840)	0.85 (1.832)	32.709^b^	<0.001
YMRS	*n* = 62	*n* = 100	*n* = 81	*n* = 129		
	2.19 (4.475)	7.69 (9.91)	1.57 (2.945)	0.16 (0.542)	21.912^b^	<0.001
BPRS	*n* = 112	*n* = 63	*n* = 44	*n* = 90		
	35.64 (13.87)	26.27 (9.378)	26.7 (6.829)	18.27 (0.761)	17.358^b^	<0.001
WCST	*n* = 59	*n* = 63	*n* = 62	*n* = 100		
Correct responses	18.03 (11.926)	23.81 (12.829)	24.18 (12.154)	29.86 (12.135)	4.702^b^	<0.001
Categories completed	1.58 (1.812)	2.79 (2.164)	2.92 (1.969)	3.89 (2.197)	8.344^b^	<0.001
Total errors	29.97 (11.926)	23.71 (12.902)	23.81 (12.156)	18.05 (12.229)	5.066^b^	<0.001
Perseverative errors	13.14 (12.542)	10.02 (10.256)	10.58 (10.647)	6.78 (7.066)	1.337^b^	<0.001
Non-perseverative errors	16.73 (8.833)	14.05 (7.705)	13.23 (6.58)	11.41 (6.796)	3.367^b^	<0.001
Mean FD	0.12 (0.06)	0.11 (0.05)	0.13 (0.06)	0.11 (0.05)	3.214^a^	0.023

Data are presented as either a number or the mean (SD).

SZ, Schizophrenia; BD, Bipolar Disorder; MDD, Major Depressive Disorder; HC, Healthy Control; HAMD-17, 17-item Hamilton Rating Scale for Depression; HAMA, Hamilton Rating Scale for Anxiety; YMRS, Young Mania Rating Scale; BPRS, Brief Psychiatric Rating Scale; WCST, Wisconsin Card Sorting Test; FD, framewise displacement.

a The comparation among the SZ, BD, MDD, and HC groups.

b The comparation among the SZ, BD, and MDD groups.

### Calculation of FC

**Figure 1:**
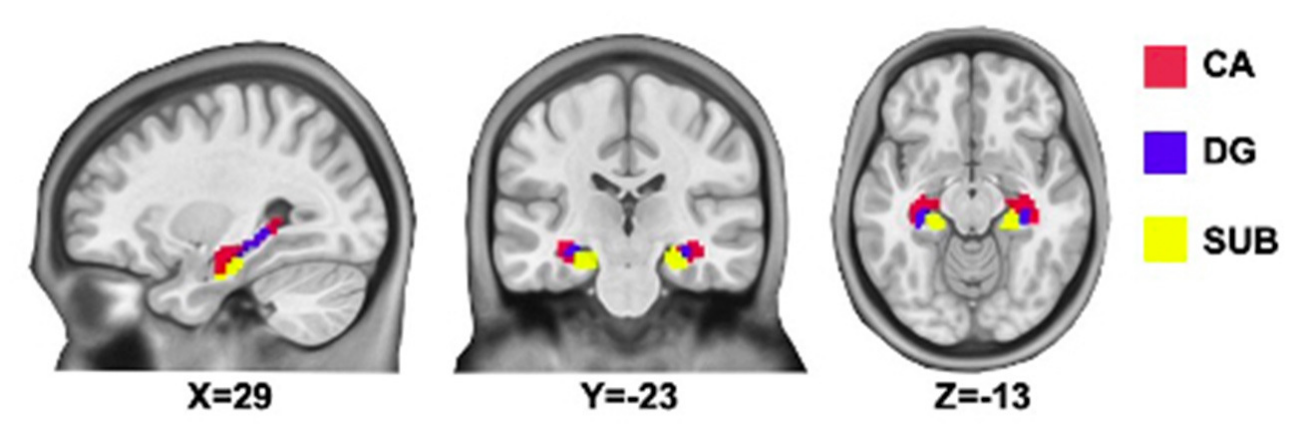
Hippocampal subregions. CA, Cornu Ammonis (shown in red); DG, Dentate Gyrus (shown in blue); SUB, subiculum (shown in yellow).

The bilateral hippocampal seed region of interest (ROI) was determined using stereotaxic, probabilistic maps of cytoarchitectonic boundaries, which included cornu ammonis (CA), dentate gyrus (DG), and subiculum (SUB) (Figure 1). The ROI was created in standard space and based on voxels with at least 50% probability of belonging to the hippocampus. For each subject, a mean time series of the hippocampal subregion seed was calculated by averaging the time series for all voxels within the ROI. Correlational analyses were then performed between the hippocampal subregion ROI time series and the time series of each brain voxel. The correlation coefficients in each map were transformed to Z values using Fisher r-to-z transformation for statistical testing.

### Statistical analyses

We performed analyses of demographic and clinical characteristics and cognitive measures using analysis of variance and χ^2^ tests. Results were considered significant at *p* < 0.05.

We performed a voxel-wise one-way analysis of covariance (ANCOVA) of each hippocampal subregion with four diagnostic groups (SZ, BD, MDD, and HC) with age, gender, years of education, and mean framewise displacement (FD) as covariates. To correct for multiple comparisons, we used Gaussian Random Field correction (GRF) (*p* < 0.005) and cluster for a corrected significant *p* < 0.05. Then, we performed *post-hoc* pair-wise *t* contrasts (SZ vs. HC, BD vs. HC, and MDD vs. HC) of each hippocampal subregion to visualize differences between each patient group and HC in regions that showed significant differences in the ANCOVA analysis. For the *post-hoc* pair-wise analyses, we used age, gender, years of education and mean FD as covariates, and GRF for correction (voxel *p* < 0.001 and cluster *p* < 0.05) following current standard (30).

To further explore the meaning of the altered FC of different hippocampal subregions, we performed partial correlation analyses (two tailed) between FC value and clinical symptoms or WCST, controlling for age, gender, years of education and mean FD. For the shared altered FC of SZ, BD, and MDD, partial correlation analyses were performed in one patient group consisting of the three diagnostic groups. For the shared altered FC of SZ and BD, partial correlation analyses were performed in one patient group consisting of the two diagnostic groups. For the specific altered FC of SZ or BD, partial correlation analyses were performed only in the SZ or BD group.

## Results

### Demographic and clinical data

Demographic and clinical characteristics are presented in Table 1. There were no significant differences in age or gender among the SZ, BD, MDD, and HC groups. Significant difference was found in years of education (*F* = 36.641, *p* < 0.001). As for clinical characteristics, significant differences were noted in HAMD-17 (*F* = 57.849, *p* < 0.001), HAMA (*F* = 32.709, *p* < 0.001), YMRS (*F* = 21.912, *p* < 0.001), and BPRS scores (*F* = 17.358, *p* < 0.001) among the SZ, BD, and MDD groups, as well as duration of disease (*F* = 6.556, *p* = 0.003), whether participants were in their first episode of illness (χ^2^ = 21.240, *p* < 0.001), and medication (χ^2^ = 6.556, *p* = 0.001). Significant differences of cognitive function among the SZ, BD, and MDD groups were also observed (correct responses: *F* = 4.702, *p* < 0.001; categories completed: *F* = 8.344, *p* < 0.001; total errors: *F* = 5.066, *p* < 0.001; perseverative errors: *F* = 1.337, *p* < 0.001; non-perseverative errors: *F* = 3.367, *p* < 0.001). A significant difference was also found in mean FD among the four groups (*F* = 3.214, *p* < 0.023).

### Functional connectivity of hippocampal subregions

#### CA subregion

**Figure 2:**
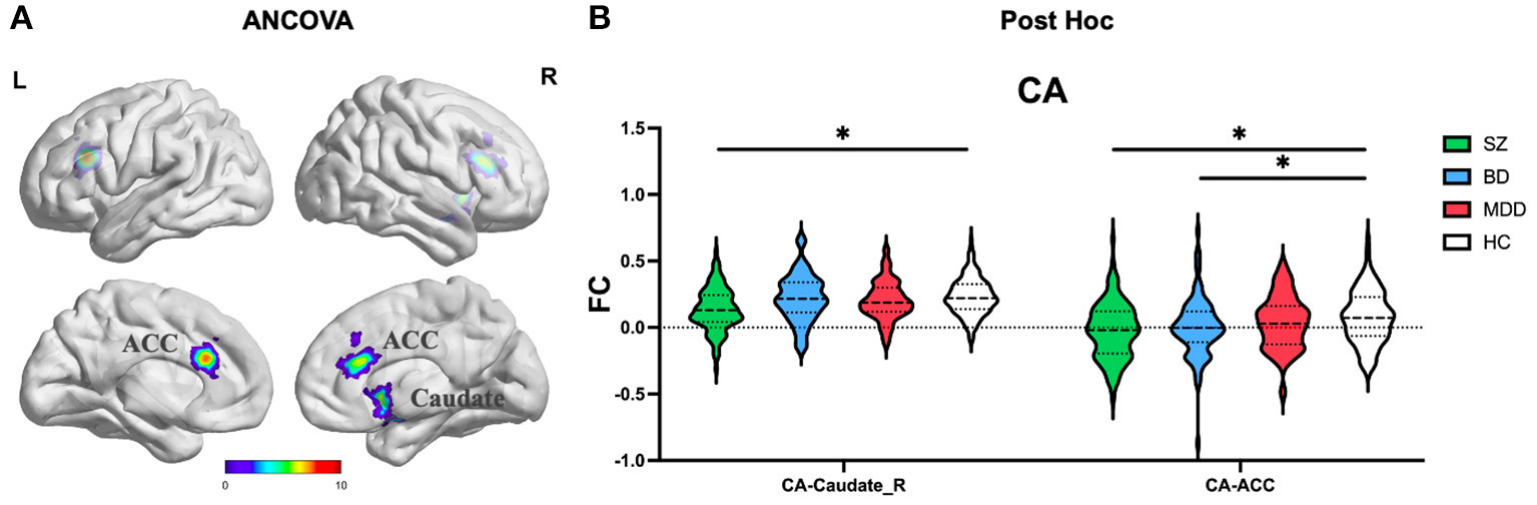
The FC alterations of CA subregion analyzed by **(A)** ANCOVA analyzed. **(B)** The *post hoc* pair-wise *t* test. FC, Functional Connectivity; CA, Cornu Ammonis; ANCOVA, Analysis of Covariance; SZ, Schizophrenia; BD, Bipolar Disorder; MDD, Major Depressive Disorder; GRF, Gaussian Random Field. ANCOVA: Significance was set at cluster *p* < 0.05 corrected using GRF with voxel *p* < 0.005. *Post-hoc*: *Significance was set at cluster *p* < 0.05 corrected using GRF with voxel *p* < 0.001.

ANCOVA of FC showed two clusters with significant group differences of CA ROI (Table 2; Figure 2A). These clusters consist of anterior cingulate cortex (ACC) and right caudate.

**Table 2:** Brain regions demonstrating significant differences in ANCOVA.

**ROI**	**Brain region**	**Cluster size**	**MNI coordinates**			
			***X***	***Y***	***Z***	***F-*** **value**
CA	Caudate_R	77	9	12	−3	7.062
	ACC	82	0	24	24	8.1925
DG	ACC	68	0	24	24	8.0583
SUB	MTG_L	178	−51	−36	−3	9.0897
	IFOG_L	128	−39	27	−6	8.5548
	MFG_R	134	33	39	24	6.7541
	SMA	128	3	15	48	6.636

ANCOVA, Analysis of Covariance; CA, Cornu Ammonis; DG, Dentate Gyrus; SUB, Subiculum; Caudate_R, Right Caudate; ACC, Anterior Cingulate Cortex; MTG_L, Left Middle Temporal Gyrus; IFOG_L, Left Inferior Orbital Frontal Gyrus; MFG_R, Right Middle Frontal Gyrus; SMA, Supplemental Motor Area.

#### DG subregion

**Figure 3:**
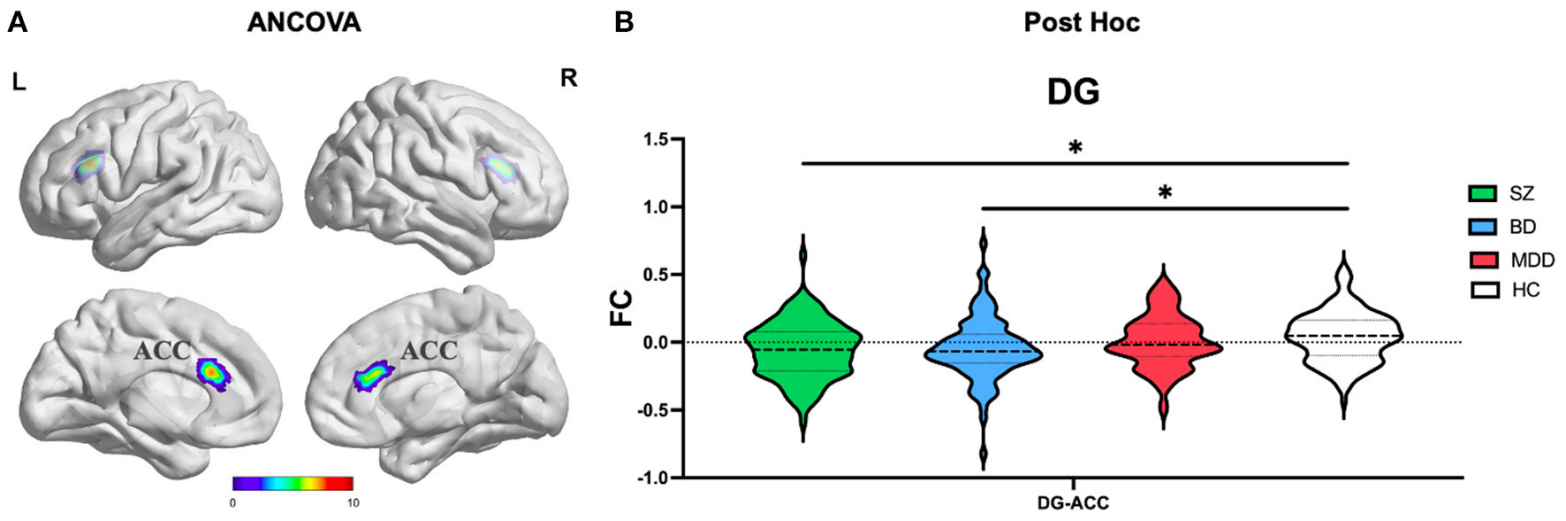
The FC alterations of DG subregion analyzed by **(A)** ANCOVA analyzed. **(B)** The *post hoc* pair-wise *t* test. FC, Functional Connectivity; DG, Dentate Gyrus; ANCOVA, Analysis of Covariance; SZ, Schizophrenia; BD, Bipolar Disorder; MDD, Major Depressive Disorder; GRF, Gaussian Random Field. ANCOVA: Significance was set at cluster *p* < 0.05 corrected using GRF with voxel *p* < 0.005. *Post-hoc*: *Significance was set at cluster *p* < 0.05 corrected using GRF with voxel *p* < 0.001.

ANCOVA of FC showed one cluster with significant group differences of DG ROI (Table 2; Figure 3A). This cluster is also in ACC.

#### SUB subregion

**Figure 4:**
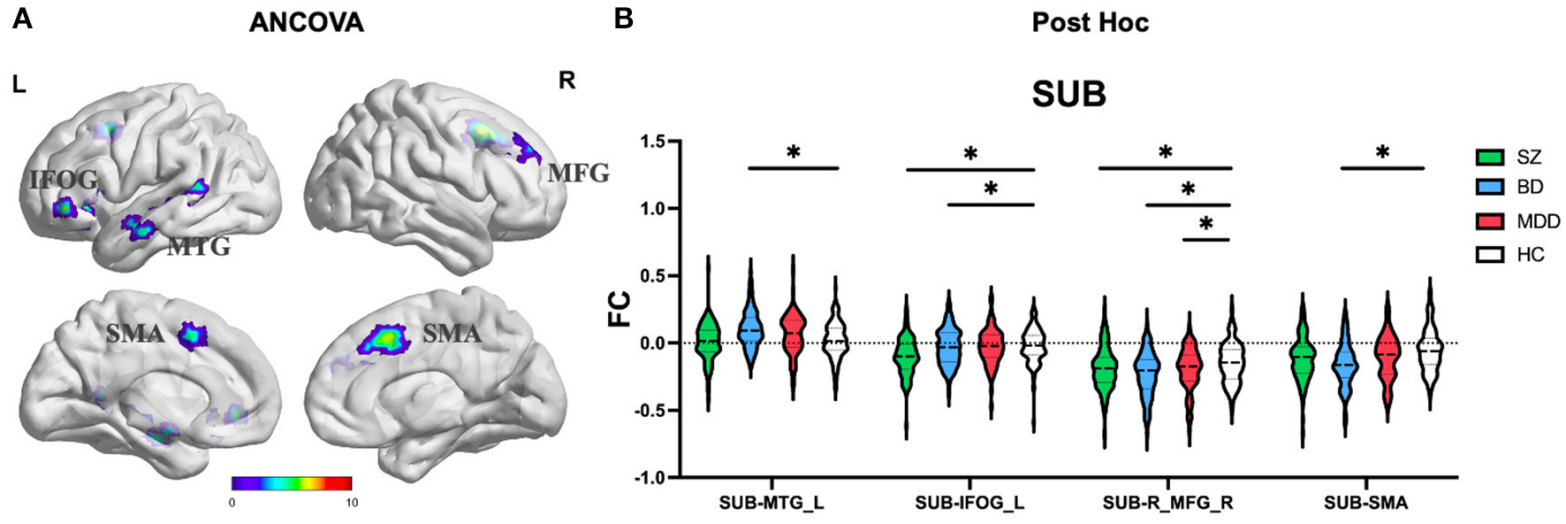
The FC alterations of SUB subregion analyzed by **(A)** ANCOVA analyzed. **(B)** The *post hoc* pair-wise *t* test. FC, Functional Connectivity; SUB, Subiculum; ANCOVA, Analysis of Covariance; SZ, Schizophrenia; BD, Bipolar Disorder; MDD, Major Depressive Disorder; GRF, Gaussian Random Field. ANCOVA: Significance was set at cluster *p* < 0.05 corrected using GRF with voxel *p* < 0.005. *Post-hoc*: *Significance was set at cluster *p* < 0.05 corrected using GRF with voxel *p* <0.001.

ANCOVA of FC showed four clusters with significant group differences of the SUB subregion (Table 2; Figure 4A). These clusters consist of left middle temporal gyrus (MTG_L), left inferior orbital frontal gyrus (IFOG_L), right middle frontal gyrus (MFG_R), and bilateral supplemental motor area (SMA).

#### Commonly and distinctly altered FC

(1) CA subregion: *Post-hoc* analyses revealed decreased FC of ACC in each of the SZ and BD groups compared with HC, but not in the MDD group (Figure 2B). As for right caudate, *post-hoc* analyses only found decreased FC in the SZ group compared with HC, but not in the BD and MDD groups. (2) DG subregion: *Post-hoc* analyses revealed decreased FC of ACC in each of the SZ and BD groups compared with HC, but not in the MDD group (Figure 3B). (3) SUB subregion: *Post-hoc* analyses revealed decreased FC of MFG_R in each of the SZ, BD, and MDD groups compared with HC (Figure 4B). In MFG_R, the order of FC values was as follows: BD < MDD = SZ < HC. In addition, *post-hoc* analyses also found significantly decreased FC of IFOG_L and in SZ and BD but not in MDD compared with HC. Furthermore, *post-hoc* analyses found increased FC of MTG_L and decreased FC of SMA only in BD group compared with HC, but not in the SZ and MDD groups.

### Partial correlation analysis between altered hippocampal FC and clinical characteristics

**Figure 5:**
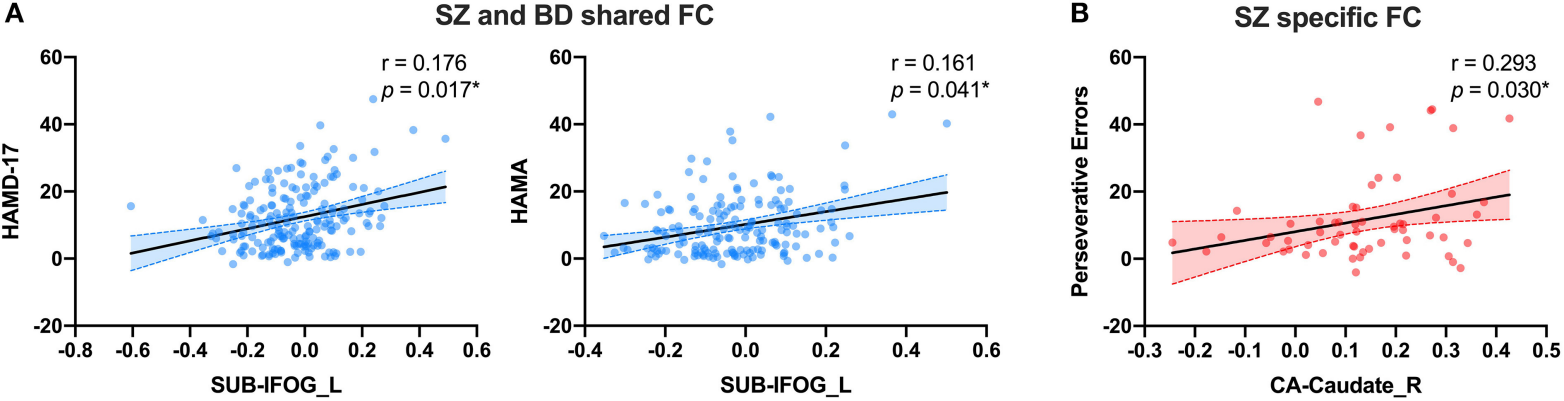
The partial correlation results of shared or distinct altered FC with clinical characteristics. **(A)** The partial correlation between SUB-MTG_L and HAMD-17 and HAMA in SZ and BD groups. **(B)** The partial correlation between CA-Caudate_R and preservative errors of WCST in SZ group. FC, Functional Connectivity; CA, Cornu Ammonis; SUB, Subiculum; SZ, Schizophrenia; BD, Bipolar Disorder; MDD, Major Depressive Disorder; HAMD-17, 17-item Hamilton Rating Scale for Depression; HAMA, Hamilton Rating Scale for Anxiety; IFOG_L, Left Inferior Orbital Frontal Gyrus; Caudate_R, Right Caudate. All the scattered points were controlled by age, gender, years of education and mean FD. *Significance was set at *p* < 0.05.

After identifying the abnormal FC of hippocampal subregions, we then investigated how they were related to clinical characteristics. For the altered FC in common among SZ, BD, and MDD, we did not found correlation between the altered FC value and clinical characteristics (HAMD-17, HAMA, YMRS, and BPRS) or cognitive function (WSCT). For the altered FC in common between BD and SZ, we found positive partial correlations between the altered FC value of SUB-IFOG_L and HAMD-17 (Figure 5A, r = 0.176, *p* = 0.017) and HAMA (Figure 5A, r = 0.161, *p* = 0.041). In addition, for the altered FC only in the SZ group, we also found a positive partial correlation between CA-Caudate_R and perseverative errors of WCST (Figure 5B, *r* = 0.293, *p* = 0.030). In the BD group, we did not find the significant correlation between SUB-MTG_L or SUB-SMA and clinical characteristics or WCST.

## Discussion

In this study, we examined the FC of three hippocampal subregions of SZ, BD, and MDD. We found commonly and distinctly changed FC of hippocampal subregions in SZ, BD, and MDD. The commonly altered FC were as follows: (1) In the SUB subregion, SZ, BD, and MDD all showed changed FC of SUB-MFG_R. We do not find the significant correlation between SUB-MFG_R and clinical symptoms or WCST. (2) Both SZ and BD showed decreased FC in CA-ACC, DG-ACC and SUB-IFOG_L, but not in MDD. And SUB-IFOG_L had a positive correlation with HAMD-17 and HAMA in these two groups. We also found distinctly changed FC of hippocampal subregions of the three psychiatric disorders: (1) Only SZ showed decreased FC in CA-caudate_R and it had a positive correlation with WCST of SZ. (2) Only BD showed increased FC in SUB-MTG_L and decreased FC in SUB-SMA. We do not find the significant correlation between SUB-MTG_L or SUB-SMA and clinical symptoms or WCST of BD.

### Shared alteration FC of hippocampal subregions across diagnostic groups

In this study, the decreased FC of SUB and MFG might be a common feature of SZ, BD, and MDD. The SUB receives direct synaptic inputs from the hippocampal CA1 area and projects to various cortical and subcortical areas, playing a crucial role in organizing hippocampal output and having a unique function in information processing (31). The frontal cortex has long been considered as the core regions regulating the pathophysiological changes in psychiatric disorders which playing a crucial role in working memory and attention control (32–36). Although the clinical manifestations in different diagnostic categories may be quite different, they may have similar intrinsic changes from the perspective of neuroimaging, which suggests the possibility of using neuroimaging to subtype and further explore neurobiological mechanisms. Recently, there have been some attempts to subtype using neuroimaging features, and the importance of the frontal cortex has been seen in both single diagnostic (37) and transdiagnostic subtyping (38). Therefore, combined with the above information, it is suggested that the frontal cortex may serve as a key feature to interpret the common and specific changes in psychiatric disorders.

Another important finding of this study is that SZ and BD share more common changes, but these changes are not found in MDD. SZ and BD both having decreased connectivity in CA-ACC, DG-ACC, and SUB-IFOG. Previous studies found that CA and DG have similar functions in coding new associations with novel information (39). ACC receives projections from the orbitofrontal cortex and provides a direct monosynaptic connection onto hippocampal pyramidal cells in the CA3/CA1 subfields with properties that mediate the retrieval of recently encoded memory traces (40, 41). In addition, ACC is considered to be an information processing center for emotion, social interaction, and cognition (42). Lesions in ACC regions have been shown to have resulted in cognition and emotional dysregulation in psychiatric disorders. Previous studies found structural and functional abnormalities of ACC in SZ and BD (43–46), suggesting that ACC might be a common core lesion underlying the psychopathology of SZ and BD. We also found that, in the functional imbalance between hippocampus and ACC, it is the CA and DG subregions, rather than the SUB, that are mainly involved in such dysfunction, which further suggests that the function of CA and DG are similar in neuroimaging mechanisms of psychiatric disorders.

For the SUB subregion, we found significantly changed FC in IFOG in SZ and BD. IFOG, which is part of the orbitofrontal cortex and crucial nodes in the frontoparietal circuit, is involved in decision-making, reward learning affective processes, and cognitive control (47, 48). Previous studies have found that the local FC of orbitofrontal cortex showed increased in SZ, BD, and MDD (10), but for the distal FC of hippocampus and orbitofrontal cortex, we did not find the same pattern in MDD, suggesting that the functional abnormalities of orbitofrontal cortex in MDD may be different from those in SZ and BD. In addition, a positive correlation between SUB-IFOG and clinical symptoms in SZ and MDD also been found, especially depressive symptom and anxiety symptom, which indicating that functional abnormality of orbitofrontal cortex might be intrinsic to the clinical manifestations of SZ and BD.

### Diagnosis-specific functional abnormalities of hippocampal subregions

In this study, we also found some disease-specific changes, mainly SZ and BD. The FC changes between the CA subregion and caudate was only found in the SZ group relative to HC. The caudate nucleus is part of the striatum, and its functions include not only planning the execution of movement, but also learning, memory, reward, motivation, emotion, and romantic interaction (49). The striatum is the home of dopamine receptors and associated with working memory, flexibility, decision-making, purposeful behavior, and learning (50). Some studies have also reported that dopamine receptors distributed in the hippocampus are related to cognitive function (51). Disrupted dopaminergic modulation of the hippocampal-striatum circuit is associated with deficits in reward and associative learning, which are core deficits in SZ (52). Previous studies found that the strength of functional connectivity of the hippocampal-striatum circuit is abnormal in SZ patients (53, 54). The abnormal FC of CA-caudate_R showed a positive correlation with WCST in the SZ group, in a cognitive function test. Thus, we speculate that our findings of altered FC between the CA subregion and caudate could be related to the function of the dopaminergic system, and such changes may in turn be related to the cognitive deficits of SZ. Furthermore, abnormal FC in the caudate may also serve as a differentiating feature of SZ.

The increased FC of SUB-MTG and decreased FC of SUB-SMA were only found in BD group. Temporal cortex is located around the hippocampal-entorhinal complex and involves in the processing of emotions and cognitions. Structural and functional abnormalities of MTG in BD have been supported by other studies (55, 56). A recent study also found that BD showed a relationship between weight gain and MTG volume loss (57). SMA lies in the superior frontal gyrus and previous studies found that it is not simply a motor structure but also subserves more “cognitive” processes (58, 59). At present, there are few reports of SMA abnormalities in BD, only one study found the FC of amygdala and SMA might be the feature of manic state of BD (60). Considering the other findings in this study, we suggest that the abnormal FC of hippocampus with MTG and SMA might be a specific alteration in differentiating BD from SZ and MDD.

## Limitations

Our study also had some limitations. First, a large proportion of our patients had received psychiatric medication, which might affect brain function. Future studies in medication-naïve patients are needed to better clarify the mechanisms by which the different hippocampal subregions are involved in the three disorders. Second, there was also a relatively wide age range in the present sample (13–45 y) and age could also affect brain function, but we used age-matched healthy controls to minimize this effect. Third, the partial correlation analyses were not adjusted for multiple comparisons, so the correlation analyses in this study was exploratory. Finally, our study is a cross-sectional study, and a longitudinal study is needed to better understand the trans-diagnostic pathophysiological mechanisms.

## Conclusions

In summary, we examined the role of different hippocampal subregions in SZ, BD, and MDD by examining FC of each subregion with the whole brain. The abnormal functions of MFG might be the core feature of the psychopathological mechanisms of SZ, BD, and MDD. ACC and IFOG might be the shared abnormalities of SZ and BD, and IFOG are also positively correlated with depression and anxiety symptoms in SZ and BD. Caudate might be the unique feature of SZ and showed a positive correlation with the cognitive function in SZ. MTG and SMA are the differentiating features of BD. Our study provides evidence for the different functions of different hippocampal subregions in psychiatric pathology.

## Data availability statement

The original contributions presented in the study are included in the article/supplementary material, further inquiries can be directed to the corresponding authors.

## Ethics statement

The studies involving human participants were reviewed and approved by the Medical Science Research Ethics Committee of the China Medical University [approval reference number (2012)25–1]. Written informed consent to participate in this study was provided by the participants' legal guardian/next of kin.

## Author contributions

YT and FW conceptualized and supervised the study. YS and JY participated in the design of the study, collection the data, statistical analysis, interpretation of the data, and the drafting of the article. MC, YW, ZY, YueZ, YunZ, YifZ, and XJ participated in the data acquisition, analyses, and interpretation of the data. FW, LK, and KX revised the manuscript and provided technical support. All authors contributed to the article and approved the manuscript submission.

## Funding

This study was funded by National Science Fund for Distinguished Young Scholars (81725005 to FW), the National Key R&D Program of China (2018YFC1311600 and 2016YFC1306900 to YT), Liaoning Revitalization Talents Program (XLYC1808036 to YT), Joint Fund of National Natural Science Foundation of China (U1808204 to FW), Natural Science Foundation of Liaoning Province (2019-MS-05 to FW), and Natural Science Foundation of Liaoning Province (2020-MS-176 to XJ).

## Conflict of interest

The authors declare that the research was conducted in the absence of any commercial or financial relationships that could be construed as a potential conflict of interest.

## Publisher's note

All claims expressed in this article are solely those of the authors and do not necessarily represent those of their affiliated organizations, or those of the publisher, the editors and the reviewers. Any product that may be evaluated in this article, or claim that may be made by its manufacturer, is not guaranteed or endorsed by the publisher.
